# Biomimetics in Botanical Gardens—Educational Trails and Guided Tours

**DOI:** 10.3390/biomimetics8030303

**Published:** 2023-07-11

**Authors:** Olga Speck, Thomas Speck

**Affiliations:** 1 Cluster of Excellence *liv*MatS@FIT—Freiburg Center for Interactive Materials and Bioinspired Technologies, University of Freiburg, Georges-Köhler-Allee 105, 79110 Freiburg, Germany; thomas.speck@biologie.uni-freiburg.de; 2 Plant Biomechanics Group@Botanic Garden Freiburg, University of Freiburg, Schänzlestr. 1, 79104 Freiburg, Germany

**Keywords:** barbed wire, botanical garden, biomimetics tour, biomimetics trail, facade shading, Lotus-Effect^®^, self-repair, technical plant stem, Velcro^®^

## Abstract

The first botanical gardens in Europe were established for the study of medicinal, poisonous, and herbal plants by students of medicine or pharmacy at universities. As the natural sciences became increasingly important in the 19th Century, botanical gardens additionally took on the role of public educational institutions. Since then, learning from living nature with the aim of developing technical applications, namely biomimetics, has played a special role in botanical gardens. Sir Joseph Paxton designed rainwater drainage channels in the roof of the Crystal Palace for the London World’s Fair in 1881, having been inspired by the South American giant water lily (*Victoria amazonica*). The development of the Lotus-Effect^®^ at the Botanical Garden Bonn was inspired by the self-cleaning leaf surfaces of the sacred lotus (*Nelumbo nucifera*). At the Botanic Garden Freiburg, a self-sealing foam coating for pneumatic systems was developed based on the self-sealing of the liana stems of the genus *Aristolochia*. Currently, botanical gardens are both research institutions and places of lifelong learning. Numerous botanical gardens provide biomimetics trails with information panels at each station for self-study and guided biomimetics tours with simple experiments to demonstrate the functional principles transferred from the biological model to the technical application. We present eight information panels suitable for setting up education about biomimetics and simple experiments to support guided garden tours about biomimetics.

## 1. Botanical Gardens through the Ages

### 1.1. The Botanical Garden—A Paradise on Earth

Botanical gardens are enclosed spaces, which, unlike wild nature outside, can be shaped according to people’s ideas, wishes, and desires. Outside, the wilderness looms, whereas in the garden protected by a high wall or fence and guarded by a gate, we find shady and fruit-bearing trees, flowerbeds, and a spring or lake. These descriptions undoubtedly correspond to the idea of a paradise on Earth or a Garden of Eden, which brings diversity and beauty into the garden with the goal of guiding meetings between humans and living nature. Etymologically, the Greek–Latin word *paradise* comes from the Old Iranian Awestic language, where *pairi daēza* stands for a walled enclosure. The Garden of Eden, also called the Terrestrial Paradise, is the biblical paradise described in the Book of Genesis. In addition, the Latin name *hortus* means garden, with *hortus conclusus* being an enclosed garden [1].

**Figure 1:**
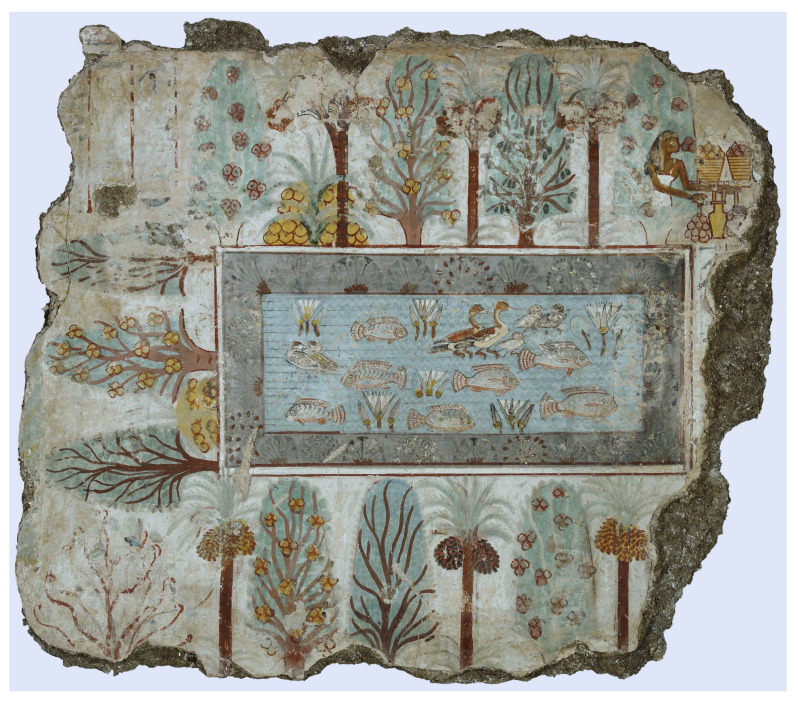
Fresco of an Egyptian garden from the Tomb of Nebamun, Thebes, ca. 1350 BCE. The rectangular pond with fish, ducks, and lotus (*Nymphaea lotus*) is framed by water plants and hedged by fruit-bearing trees such as date palms, sycamore figs, and mandrakes (reprinted under public domain from Wikimedia Common, British Museum).

Figure 1 shows a famous Egyptian fresco of a garden with a central pond surrounded by water plants and hedged by trees [2]. The parable of paradise as a garden runs throughout the occidental Middle Ages until well into the Renaissance. During the Renaissance, scientific interest in the Plant Kingdom increased, initially for medicinal reasons [1].

### 1.2. First Botanical Gardens in Europe

**Figure 2:**
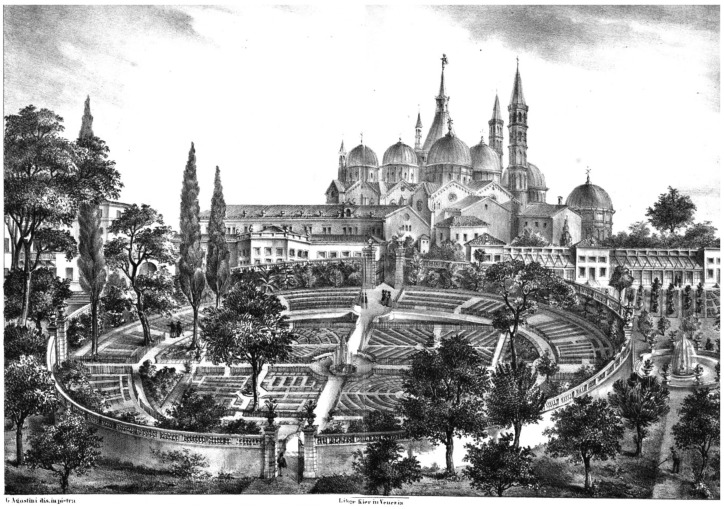
The Botanical Garden of Padova (or *Garden of the Simples*) in a 16th-Century print. In the background is the Basilica of Sant’Antonio (reprinted under public domain from Wikimedia Common).

Among the oldest botanical gardens in the world are those in Padova and Pisa [3], both founded in 1545 as herb gardens specializing in medicinal plants (*simples gardens*) in Italy. The Botanical Garden of Padova (Figure 2) was established on 1 July 1545. It belongs to the University of Padova and is still in its original location. The late Georgina Masson wrote: “The Orto Botanico at Padova was attached to the school of botany at the university, where the first chair of that science had been created shortly before…” [4]. The center of the Botanical Garden of Padova is a square within a circle, divided into four smaller squares by two main intersecting paths. The circular garden (*hortus sphairicus*) represents the paradise motif and has a central fountain surrounded by a ring of water representing the ocean (*hortus cinctus*) [4,5].

Garbari [3] refers to a letter dated on 4 July 1545, in which the great naturalist, herbalist, and physician Luca Ghini (1490–1556) writes that he had collected plants “…which I have planted in a garden of Pisa to be useful for the students…”. Ghini, who had been brought from Bologna to Pisa by Grand Duke Cosimo I of the Medici family in 1543, apparently had a piece of land at his disposal specifically for the teaching of botany. This first garden was soon replaced by a second and later by a third botanical garden at other locations in Pisa. The third garden was rectangular and followed the typical rules of an Italian Renaissance garden. It consisted of 2 × 4 large planting beds, each divided into squares, rectangles, triangles, circles, or other odd symmetries. Each of the eight beds had a round or octagonal fountain at the center.

**Figure 3:**
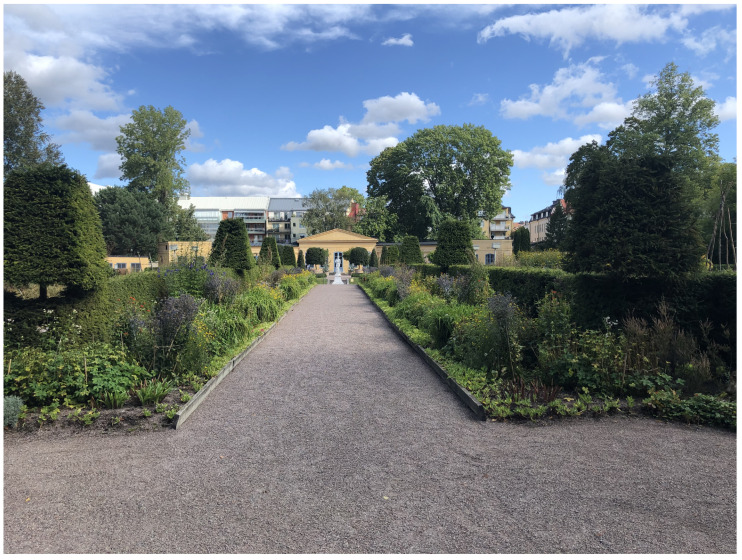
The Linnean Garden in Uppsala (Sweden). The yellow building in the background is the Orangery with the river pond (left), the lake pond (middle), and the marsh pond (right) in front of the building. Trees and shrubs to the left and right flank the rectangular planting beds (center), which have perennials and annuals, with a distinction being made between spring and autumn flowering plants.

In Germany, early botanical gardens were founded in the cities of Leipzig (1580), Jena (1586), Heidelberg (1597), Gießen (1609), and Freiburg (1620) [6,7]. Although the list of important botanical gardens in Europe, as given in the order of the year of their first appearance, e.g., with The Linnaean Garden in Uppsala, Sweden (1655), shown in Figure 3, and The Royal Botanic Gardens in Kew, United Kingdom (1759), is long, we will focus on the aspect of their changing tasks and research topics over time.

### 1.3. Botanical Gardens—Places of Learning, Research, and Relaxation

The transfer of botanical knowledge to other aspects of interest has played a central role since the foundation of the first European botanical gardens affiliated with universities. It began with the cultivation of medicinal herbs, which provided illustrative material for medical and pharmacy students to study plants. Later, botanical gardens were opened to all interested visitors for their edification. Today, botanical gardens are described as public institutions that cultivate documented living plant collections for the fulfillment of tasks in scientific research and teaching, education, sustainable preservation of plant diversity, and cultivation of plants threatened by extinction (translated from [8] by the authors). Thus, botanical gardens are currently places for learning, research, conservation, and relaxation.

Taking the Botanic Garden of the University of Freiburg, Germany, as an example, we will discuss the influence of the respective directors and their fields of science on research and education, and on the design of the garden. The Botanic Garden Freiburg was founded in 1620 and has changed its location several times. The first director, Jacobus Walter, a Professor at the Faculty of Medicine, established a *hortus medicus* to teach students of medicine. The first garden was completely destroyed by the Thirty Years’ War and the construction of the Vauban fortifications by the French in 1677. Today, the Freiburg City Garden is located at or close by the original location of the first garden in the Neuburg district.

In 1766, Franz Josua Lambert Baader, Professor of Botany and Chemistry at the Faculty of Medicine, established a new garden on the river Dreisam. Inspired by the rise of the natural sciences in general and botany in particular, he created a systematic garden with the aim of showing the relationships of plants based on morphological characteristics. Baader and other garden directors transformed the second garden from a teaching institution for aspiring medical doctors and pharmacists into a scientific research institution in its own right. The second garden had to be abandoned because of the many floods caused by the river Dreisam and the city expansion of Freiburg.

In 1879, the third Botanic Garden Freiburg was established in today’s Institute Quarter as a garden complex with modern greenhouses, beds to illustrate the latest plant classification system, and an arboretum. Director Johann Friedrich Oltmanns, Professor of Botany, opened the Botanic Garden Freiburg for the first time to both members of the university and public visitors. The original site of the third garden still has many old trees from the time when the green areas between the current buildings of the Chemical and Physical Institutes formed a botanical garden.

**Figure 4:**
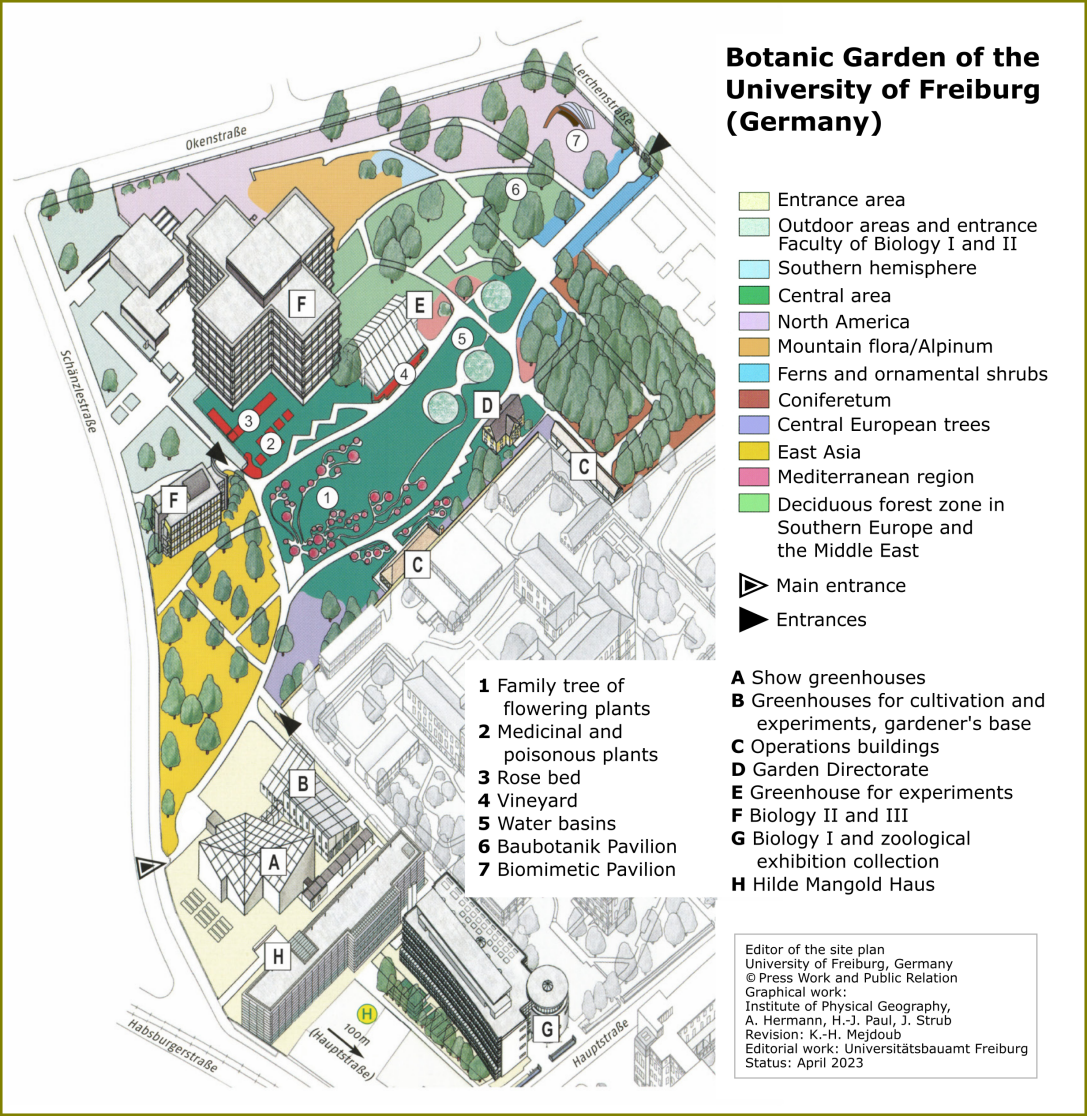
Site plan of the Botanic Garden of the University of Freiburg. The garden was founded in 1912 as a geographical garden. In the 1960s, a walk-through evolutionary family tree (1) was added at the center. It represents the historic systematic tree of plants as accepted before the arrival of molecular phylogeny. In the 2000s, the educational trail about biomimetics was established throughout the garden, with the biomimetic pavilion (7) being added in 2021. The gardener’s base (B) was equipped with the biomimetic facade shading systems Flectofin^®^ and FlectoLine in 2023. Based on the Flectofold, the FlectoLine facade shading system was developed by replacing the curved folds with straight actuators, making the system less expensive and easier to produce (image reprinted with permission of the University of Freiburg, Germany).

In association with the construction of the Biology Institute building, the Botanic Garden Freiburg moved to its current location in the Herdern district (Figure 4). From 1912 onward, Director Johann Friedrich Oltmanns established a plant-geographical garden taking into account the spirit of the age of travel to foreign countries. In this *hortus geographicus*, plants from the Mediterranean region, various mountainous regions (alpinum), East Asia, and North America were cultivated to the present day. In 1965, the Biology Professor and palaeobotanist Dieter Vogellehner implemented a new scientific concept with a focus on evolution. Co-author, Thomas Speck, has been Professor of Botany and Director of the Botanic Garden Freiburg since 2002. One of his research focuses is biomimetics, which is reflected in the educational trail and the guided tours about biomimetics for students and the interested public.

## 2. Botanical Gardens—A Treasure Trove of Biomimetics

**Figure 5:**
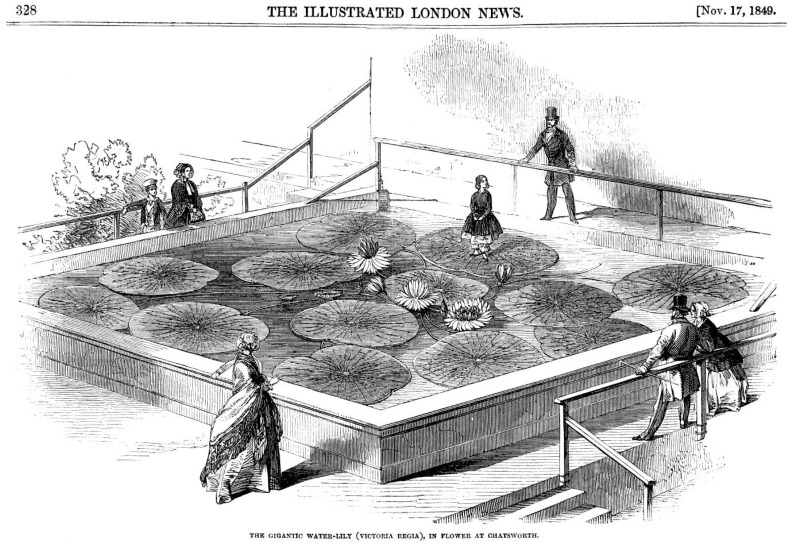
In November 1849, Sir Joseph Paxton succeeded in recreating the warm and marshy habitat of *Victoria regia*, as it was then called, in a greenhouse belonging to the Duke of Devonshire, England. The drawing shows Paxton’s daughter Anni standing on a leaf of *Victoria amazonica* in the Lily House at Chatsworth House (reprinted under public domain from Wikimedia Common).

Since the 19th Century, biomimetics, or learning from living nature with the goal of developing technical applications, has played a special role in botanical gardens. A classic example of biomimetics in architecture is the system of drainage channels for rainwater and condensation in the roof structure of the Crystal Palace, designed by the gardener and engineer Sir Joseph Paxton (1803–1865) for the Great Exhibition in London in 1881. These so-called Paxton gutters were inspired by the drainage structures on the leaves of the South American giant water lily (*Victoria amazonica*). Figure 5 shows a contemporary drawing published in the *Illustrated London News* on 17 November 1849. Sir Joseph Paxton was head gardener at Chatsworth House, owned by the Duke of Devonshire, who presented Queen Victoria with the first flowers of the plant, naming it *Victoria regia* in her honor.

The well-known biomimetic Lotus-Effect^®^ has its origins in the Botanical Garden of the University of Bonn (Germany). The development of technical surfaces with the Lotus-Effect^®^ was inspired by the self-cleaning leaf surfaces of the iconic sacred lotus (*Nelumbo nucifera*) after which it was named (Section 4.2). Several other biomimetic developments originated at the Botanic Garden Freiburg. Basic biological studies of the giant reed (*Arundo donax*) and various species of horsetail (*Equisetum hyemale*, *Equisetum giganteum*) and bamboo formed the basis for the development of the so-called “Technical Plant Stem” (Section 4.5). The model for the development of a self-sealing foam coating for pneumatic systems was the self-sealing mechanism found in the stems of lianas of the genus *Aristolochia* (Section 4.6). The hinge-less facade shading element Flectofin^®^ was inspired by the deformation of the bird-of-paradise flower (*Strelitzia reginae*) (Section 4.7), and the shading system Flectofold was inspired by the folding of the underwater carnivorous plant *Aldrovanda vesiculosa* (Section 4.8).

## 3. Lifelong Learning in Botanical Gardens

Out-of-school places such as science centers with their educational activities, science days where scientists answer questions about their research, and botanical gardens with their green schools are perfect places of lifelong learning. Active participation in social life and the higher demands of the labor market require more than the “stock of knowledge” acquired at school; this “stock” needs to be extended, deepened, and supplemented throughout life.

The scientific discipline of biomimetics is a completely new interdisciplinary field of research that has found its way into several botanical gardens in recent years. Biomimetics, i.e., learning from living nature for developing technical applications [9,10], is also reflected in the educational motivations of several botanical gardens in the form of educational trails with information boards about the biological model, the transferred functional principle, and the biomimetic application at each station (Section 4). In addition, scientists and gardeners offer guided biomimetics tours for interested visitors of all ages, using simple experiments to illustrate the relevant functional principle (Section 5). The portfolio is completed by public lectures on various biomimetics topics, scientific and popular science publications, and the development of biomimetics teaching modules for kindergarten children, pupils, and high school, college, and university students via print media and the Internet [11,12,13,14].

Some botanical gardens offer these activities on a permanent basis (Table 1), whereas others include the topic of biomimetics in their programs on special occasions. One such occasion was the “Woche der Botanischen Gärten” (i.e., “Week of Botanical Gardens”) during 2011 in Germany, Austria, and the German-speaking part of Switzerland. Initiated by the Botanic Garden Freiburg, an exhibition with the theme “Was die Technik von Pflanzen lernen kann—Bionik in Botanischen Gärten” (i.e., “What technology can learn from plants—Biomimetics in botanical gardens”) was presented in 35 botanical gardens that are members of the Verband Botanischer Gärten e.V. (i.e., Association of Botanical Gardens) [15]. In addition to the poster exhibition, each garden offered its own educational programs, such as guided tours through the exhibition, lectures, and musical events or programs for children, students, and interested visitors.

**Figure 6:**
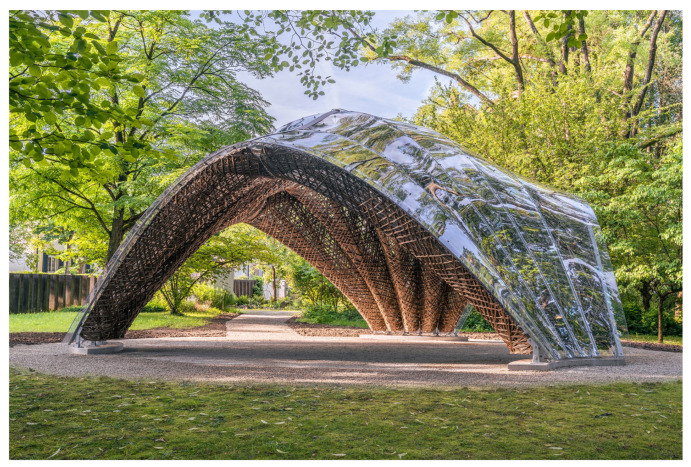
Biomimetic pavilion installed in the Botanic Garden of the University of Freiburg in 2021. The robotically manufactured flax/sisal components were inspired by the wooden cores in the trunks of various columnar cacti and prickly pears (reprinted with permission from © ICD/ITKE/IntCDC University of Stuttgart, Rob Faulkner).

Existing educational trails and guided tours about biomimetics can be supplemented at any time with current plant-inspired innovations. A prime example is the installation of the cactus-inspired pavilion in the Botanic Garden Freiburg [24,25]. The pavilion was developed and built by architects and civil engineers of the University of Stuttgart, Germany, in collaboration with biologists from the University of Freiburg. Figure 6 presents the biomimetic pavilion, which was inspired by the wooden cores of the pear cactus cladodes (*Opuntia* sp.) and the stems of the saguaro cactus (*Carnegia gigantea*), both of which show a net-like structure of fused wood strands. The load-bearing structure of the pavilion is made up of 15 flax fiber components, each between 4.50 and 5.50 m long and weighing on average 105 kg. In total, the pavilion weighs approximately 1.5 t and covers an area of 46 m^2^. Inspired by the net-like structure of the hollow cactus cores, the 15 load-bearing components and the central fibrous keystone are computer-designed and entirely made of robotically wound flax strands with sisal cords, a completely natural, renewable, and biodegradable material. The transparent ceiling is composed of poly-carbonate, which can be shredded and remelted at the end of its lifetime. Only the resin used for impregnating the flax/sisal strands could not entirely be made of bio-based material components at the time that the pavilion was built, a challenge that will be solved in the near future by lignin-based resins. The biomimetic pavilion thus demonstrates the use of natural materials and advanced digital technology in the creation of a sustainable building inspired by biological models [25].

## 4. Educational Trail about Biomimetics

In this section, we describe the educational trail about biomimetics that we developed for the outdoor area of the Botanic Garden Freiburg in 2011 as part of the “Woche der Botanischen Gärten” (i.e., Week of the Botanical Gardens). Over the last few years, we have continually added stations to the trail. The various stations present the success stories of biomimetics in the form of short portraits. Each short portrait is divided into four sections: (i) “Plant Model” describes the biological archetype, (ii) “Biomimetic Product” presents the transfer into technology, (iii) “Frequently Asked Questions” provides additional information in the context of the biomimetic development, and (iv) “Contributions to Sustainable Development” gives an impression of the impact of the biomimetic product on the environment. The texts and images are suitable templates for use on the panels of any educational trail about biomimetics. For this reason, we have kept the texts short. Where there is a need for further insight into the respective biomimetic product development, we have included a selection of key publications.

### 4.1. Biomimetics—What Is It All about?

#### 4.1.1. Learning from Living Nature

In recent years, biologists, physicists, chemists, mathematicians, engineers, material scientists, architects, and computer scientists have been working closely together to learn from nature’s functional principles and problem solutions. “Learning from living nature for developing technical applications”, also called biomimetics, always involves (i) the systematic search for suitable biological models, (ii) the deciphering of the functional principles, and (iii) the transfer of the knowledge to technical products [9].

#### 4.1.2. How to Perform Biomimetics?

**Figure 7:**
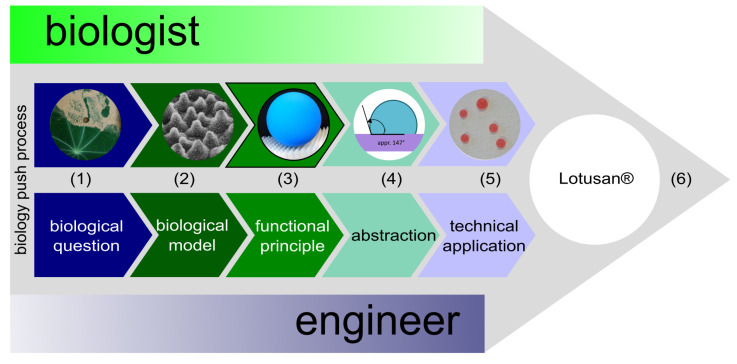
Biology push process (=biomimetic bottom-up approach) of the self-cleaning paint Lotusan^®^. (1) What is the basis of the self-cleaning effect of lotus leaves (*Nelumbo nucifera*)?; (2) SEM image showing a micro- and nano-rough plant surface equipped with wax crystalloids (photo courtesy of C. Neinhuis, TU Dresden); (3) minimal contact area between dirt particles and the surface based on a hierarchically microstructured and nanostructured and water-repellent surface in combination with water droplets; (4) lotus surfaces show contact angles of approximately 147°; (5) technical applications with this functional principle bear the trademark Lotus-Effect^®^; (6) the self-cleaning facade paint Lotusan^®^ has been on the market since 1999 (image reprinted under Creative Commons Attribution 3.0 International License from [26]).

**Figure 8:**
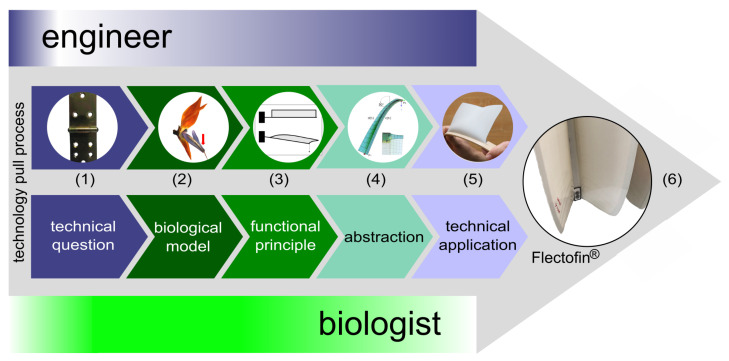
Technology pull process (=biomimetic top-down approach) of the Flectofin^®^ facade shading system. (1) How can a hinge-less motile shading system be created?; (2) hinge-less deformation of the perch of the bird-of-paradise flower; (3) lateral-torsional buckling; (4) finite element model (photo courtesy of Simon Schleicher); (5) simple physical model; (6) Flectofin^®^ facade shading system (image reprinted under Creative Commons Attribution 4.0 International License from [11]).

Basically, we distinguish two biomimetic approaches [9,10]. In the biomimetic bottom-up process (=biology push process), a technical product is developed on the basis of a functional principle found in basic biological research (Figure 7). In the biomimetic top-down approach, also known as the technology pull process (Figure 8), biomimetic improvements are sought for a functioning technical product.

#### 4.1.3. Contributions to Sustainable Development

- Biomimetics is the systematic transfer of biological knowledge to artificial products [9].
- Biomimetics creates knowledge about the common functional principles in living nature and inanimate technology [27].
- Biomimetics raises awareness of biodiversity [28,29].
- Biomimetics is not a guarantee, but an opportunity for sustainable solutions [26,29,30].

### 4.2. Lotus-Effect^®^—As Clean as a Lotus Leaf

#### 4.2.1. Plant Model: Lotus Leaf

**Figure 9:**
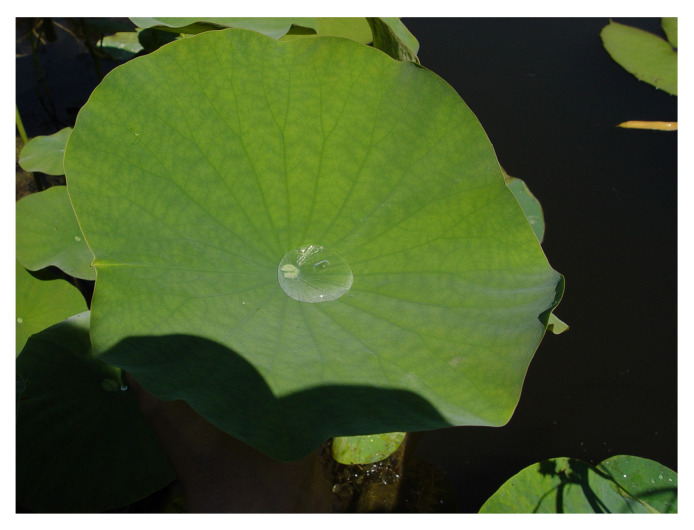
A spherical drop of water has formed on the leaf of the Indian lotus (*Nelumbo nucifera*).

**Figure 10:**
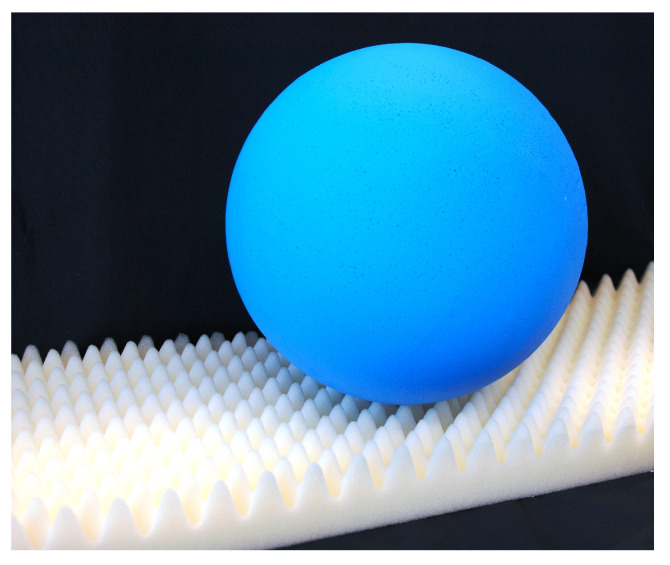
In the physical model, a spherical water droplet (blue) touches the rough self-cleaning surface (white) at only a few points and rolls up under slight inclination and vibration.

The Indian lotus (*Nelumbo nucifera*) is a symbol of purity in Hinduism and Buddhism. Although it grows in muddy ponds, its leaves are always spotlessly clean (Figure 9). It is difficult for dirt and water to adhere to the micro- and nano-roughened and water-repellent surface of the leaves. Water droplets roll off the leaf under the slightest vibrations, taking the dirt with them (Figure 10). This self-cleaning effect protects the leaves from infestation by micro-organisms such as fungi or bacteria and improves photosynthesis by removing dirt.

#### 4.2.2. Biomimetic Product: Self-Cleaning Surfaces

The botanists Wilhelm Barthlott and Christoph Neinhuis deciphered the underlying functional principle of the self-cleaning of plant surfaces during their time at the Botanical Garden in Bonn, Germany [31]. The transfer of this principle to technical surfaces led to products that are sold worldwide under the brand name Lotus-Effect^®^ [27,32]. Self-cleaning has been successfully applied to facade paints (Figure 7) and can also be found on the glass covers of the sensors in the truck toll system on German motorways.

#### 4.2.3. Frequently Asked Question: Lotus—What Is It?

The term “lotus” has many meanings in botany. On the one hand, *Lotus* is a genus of the plant family Fabaceae, which includes most bird’s-foot trefoils, such as *Lotus alpinus*. In addition, the genus *Nelumbo* is commonly called the lotus, and the water lily species *Nymphaea lotus* is also known as the tiger lotus.

#### 4.2.4. Contributions to Sustainable Development

- Plant surfaces are protected from infestation by micro-organisms by the self-cleaning function [27].
- So-called “self”-cleaning surfaces in nature and technology require water for cleaning [27,33].
- The self-cleaning facade paint Lotusan^®^ is a cost-effective and resource-saving product [34].

### 4.3. Barbed Wire–Defensive Like Plants

#### 4.3.1. Plant Model: Osage Orange

**Figure 11:**
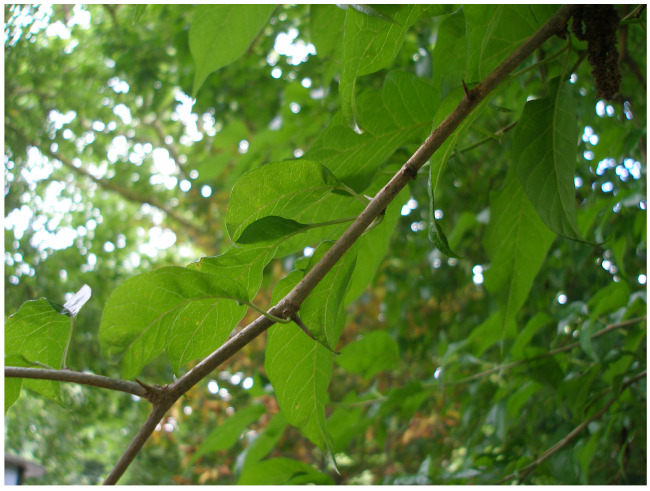
The branches of the Osage orange tree (*Maclura pomifera*) bear thorns.

One of the ways that plants defend themselves against herbivores is by thorns and spines. The Osage orange (*Maclura pomifera*) is a thorny tree (Figure 11) that was once planted by Native Americans and American farmers as hedges around cattle pastures [35].

#### 4.3.2. Biomimetic Product: Barbed Wire

**Figure 12:**
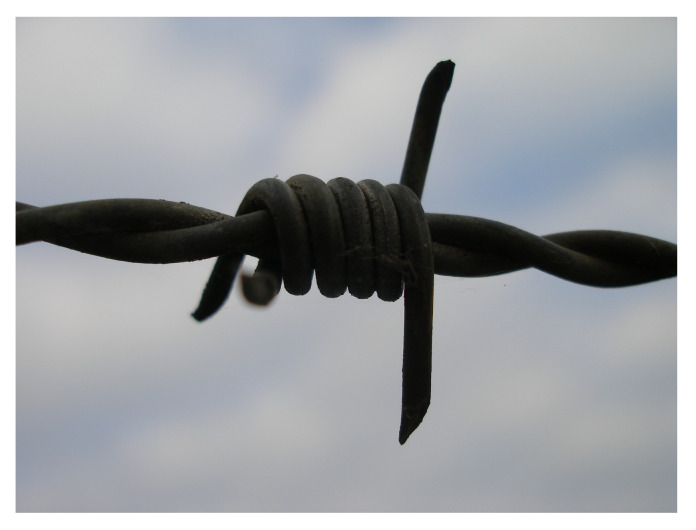
Barbed wire consists of two twisted metal wires with protruding sharp-edged tips.

In 1867, the American Lucien B. Smith invented and patented the first barbed wire, inspired by the thorny branches of the Osage orange tree. It consisted of wire with small pointed wooden pieces attached to it. In 1873, three American entrepreneurs patented barbed wire, which was made entirely of metal (Figure 12) and was much easier and less expensive to produce [36,37].

#### 4.3.3. Frequently Asked Question: Where Does the Name Osage Orange Come from?

Like the mulberry trees (genus *Morus*) and the rubber trees (genus *Ficus*), the Osage orange belongs to the mulberry family (Moraceae). From a distance, its fruits resemble an orange. The original distribution area of the Osage orange was in the south of the USA, where the Osage Native Americans settled and after whom the tree was named [35].

#### 4.3.4. Contributions to Sustainable Development

- The invention of barbed wire had both positive and negative social effects.
- Cattle ranchers and farmers used barbed wire to protect their pastures and fields [38].
- Barbed wire was an inexpensive way to control the movement of cattle, making many cowboys redundant and keeping them out of work [38].
- The—sometimes illegal—fencing of large areas of land with barbed wire deprived small ranchers and Native Americans of their land [38].
- Barbed wire is a symbol of oppression and has been used in wars [37].

### 4.4. Velcro^®^—Bonding without Adhesives

#### 4.4.1. Plant Models: Burr and Animal Fur

**Figure 13:**
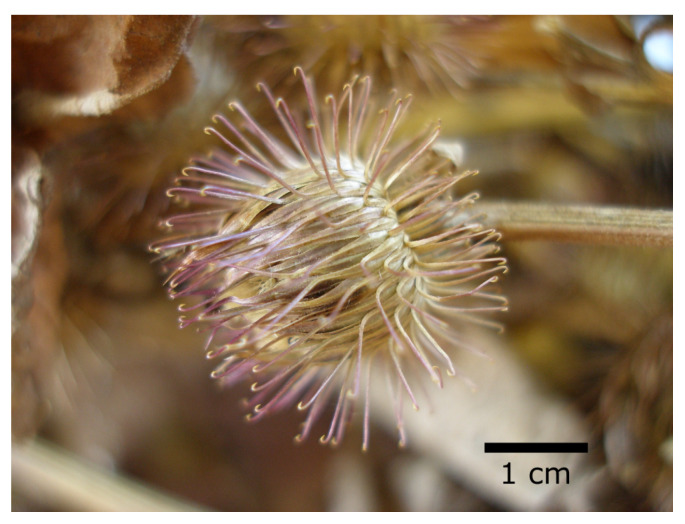
Burrs of the lesser burdock (*Arctium minus*) with hooked bracts that can attach to animal fur.

The engineer George de Mestral liked to go hunting with his dog. In the evening, he found many burdock burrs on his clothes and in his dog’s fur. He examined the burrs and discovered the small hooks that cover them (Figure 13). The hooks are so elastic that they do not break even when pulled out of the fur or removed from clothing [39].

#### 4.4.2. Biomimetic Product: Hook-and-Loop Fastener (Velcro^®^)

**Figure 14:**
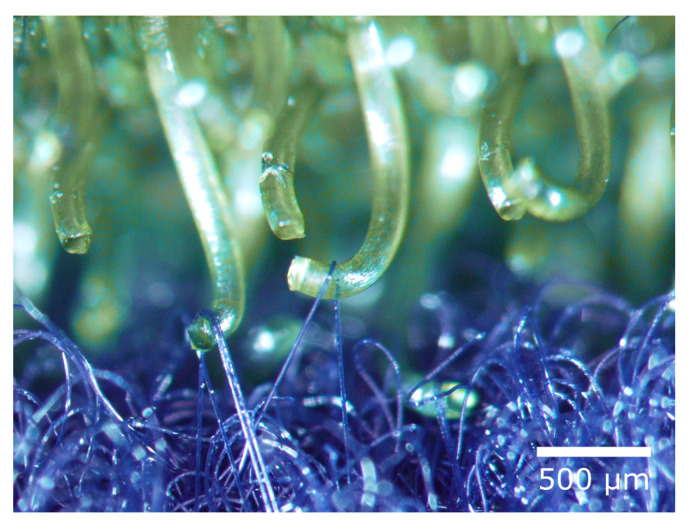
Hook-and-loop fastener (Velcro^®^) with hook tape (top) and loop tape (bottom).

Inspired by the attachment of burrs to animal fur, George de Mestral developed the hook-and-loop fastener (Velcro^®^) (Figure 14) within eight years after discovering the principle of reversible attachment. He applied for a patent for his invention in Switzerland in 1951. The hook-and-loop fastener consists of a hook tape and a loop tape. The hook tape, like the burr, has a large number of elastic hooks. The loop tape resembles animal fur and consists of many fine closed loops that can become caught in the hooks. Today, the hook-and-loop fastener is one of the best-known and commercially successful biomimetic products [40,41,42].

#### 4.4.3. Frequently Asked Question: Where Do the Hooks Come from?

Over the course of evolution, a wide variety of plant structures have developed hooks that serve to disperse fruits and the seeds that they contain. The hooks of the colewort burr (*Geum urbanum*) are transformed pistils; the burdock (*Arctium* sp.) has hooked bracts; the hooks of medick burrs (*Medicago* sp.) are formed by spines on the carpel.

#### 4.4.4. Contributions to Sustainable Development

- The reversible adhesion mechanism contributes to resource conservation by allowing repeated use and by extending the lifetime of artificial products.
- Bonding without adhesives helps to protect the environment by avoiding environmentally harmful substances.
- Understanding the propagation mechanisms of plants is a source of ideas for further technical solutions [43].

### 4.5. Technical Plant Stem—Stable and Lightweight

#### 4.5.1. Plant Models: Giant Reed, Bamboo, and Horsetail

**Figure 15:**
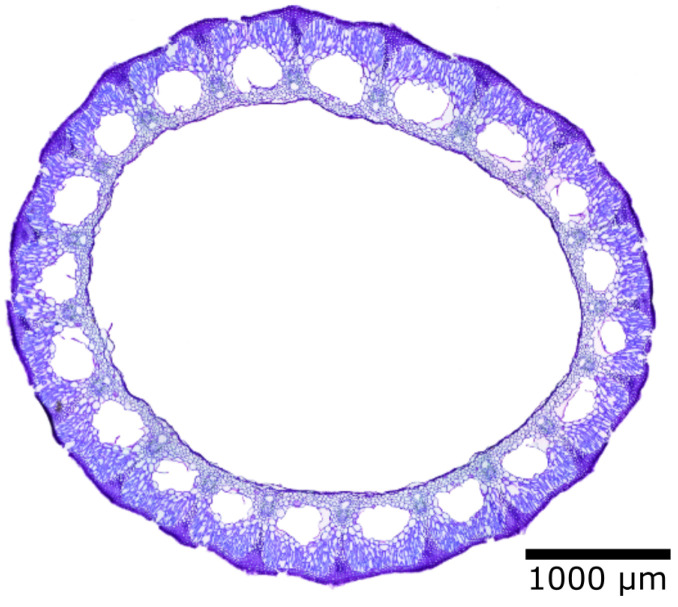
Cross-section of a winter horsetail (*Equisetum hyemale*) under a light microscope.

Horsetail, bamboo, and giant reed, with their hollow stems and thin stem walls, are both light and stable. Figure 15 shows the cross-section of the winter horsetail (*Equisetum hyemale*) under a microscope. It is obviously a lightweight construction, because even the thin stem wall of the horsetail has channels running through it [44].

#### 4.5.2. Biomimetic Product: The “Technical Plant Stem”

**Figure 16:**
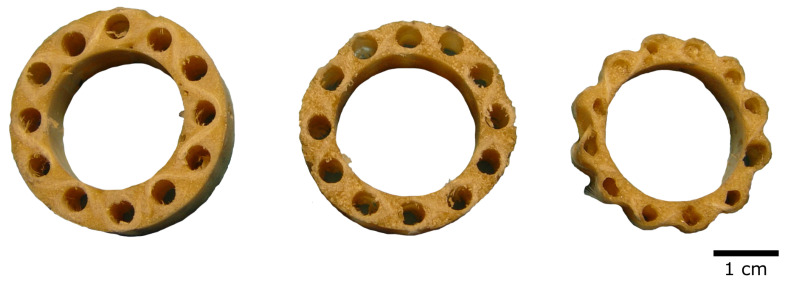
“Technical plant stem” in various versions, such as single- or double-braid and circular or star-shaped outline.

Biologists from the Botanic Garden Freiburg, together with engineers from the Deutsche Institute für Faserforschung in Denkendorf have developed and patented the “technical plant stem” (Figure 16). Inspired by horsetail, bamboo, and giant reed, it combines lightweight construction with mechanical robustness [45].

#### 4.5.3. Frequently Asked Question: Why Is the Giant Reed Also Called the Clarinet Reed?

The giant reed (*Arundo donax*) is a woody grass that can grow up to six meters high. It forms dense stands, for example, in the Camargue in southern France, where it is often planted as a windbreak. The giant reed is also called the clarinet reed because the reeds for woodwind instruments such as clarinets, oboes, saxophones, and bagpipes are made from its stem [46].

#### 4.5.4. Contributions to Sustainable Development

- Its lightweight construction conserves resources.
- The technical plant stem is fully recyclable when made from natural fibers in a matrix of natural materials.
- Damage-resistant components have a longer service life, contributing to sustainability through reduced waste generation [47].

### 4.6. Self-Repair—Not a Privilege of Nature

#### 4.6.1. Plant Model: Dutchman’s Pipe

**Figure 17:**
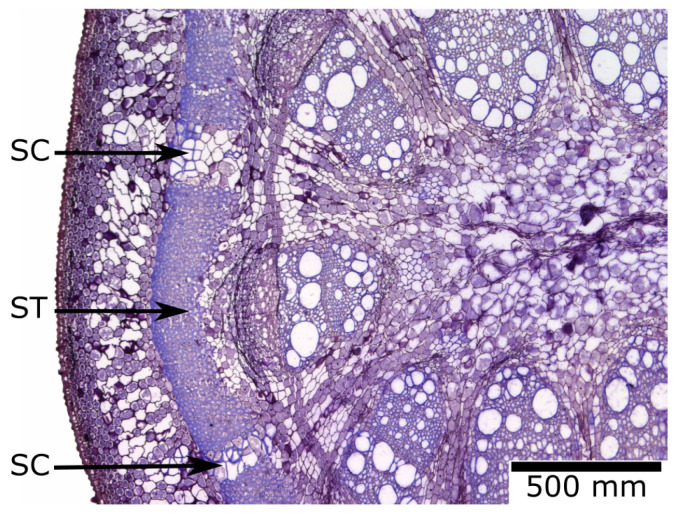
Microscopic cross-section through the stem of a Dutchman’s pipe (*Aristolochia macrophylla*) with sealing cells (SCs) closing the cracks in the outer ring of the strengthening tissue (ST) formed during the secondary growth of the inner tissues.

Over the course of biological evolution, plants have developed a variety of mechanisms to seal rapidly and subsequently to heal external and internal injuries. In the stems of the Dutchman’s pipe (*Aristolochia macrophylla*), a North American liana, rapid wound sealing occurs by sealing cells swelling into the cracks that form in the outer ring of the strengthening tissue during secondary growth (Figure 17) [48].

#### 4.6.2. Biomimetic Product: Self-Sealing Foam Coating

The principle of rapid wound sealing has been applied to a biomimetic foam coating for pneumatic systems, such as inflatable boats or Tensairity® structures. Any pressure drop is either stopped completely or is at least reduced by a factor of 100 to 1000 [49,50].

#### 4.6.3. Frequently Asked Question: Are There More Self-Repairing Materials Inspired by Plants?

**Figure 18:**
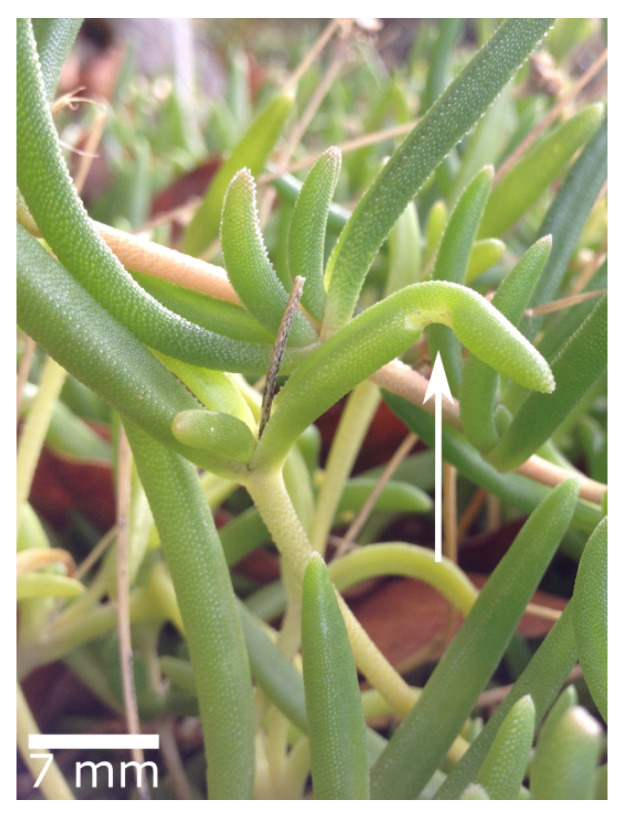
The wound-induced bending deformation (arrow) of the succulent leaves of the ice plant (*Delosperma cooperi*) has been a model for self-repairing material systems.

Wound sealing and healing have evolved many times independently in the Plant Kingdom. The various repair principles are sources of inspiration for engineering materials with self-repairing functions. For example, the wound reaction of the ice plant *Delosperma cooperi* (Figure 18) has been a model for a multi-layered actuator [51] and a polymer-based material with a shape memory effect that can heal cracks within 60 min [52].

#### 4.6.4. Contributions to Sustainable Development

- The self-repairing function of products saves resources [53].
- Products with a self-repairing function have a longer service time and lifetime [53].
- The self-repairing function of products contributes to reduced waste generation [53].

### 4.7. Flectofin^®^—Deformable without Joints

#### 4.7.1. Plant Model: The Bird-of-Paradise Flower

**Figure 19:**
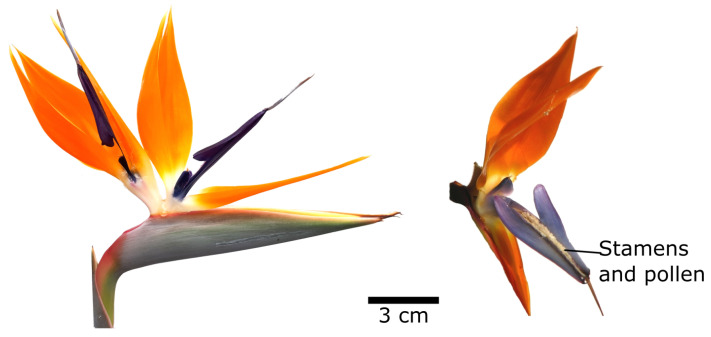
Inflorescence of the bird-of-paradise flower (*Strelitzia reginae*) with two flowers (**left**). Each flower, with its orange petals, has a purple perch, which allows the landing of pollinating nectar and weaver birds. The weight of the pollinator pushes the perch down and opens it sideways, exposing the stamens and pollen (**right**).

Plant movements are often overlooked because plants are tied to their location and because the movements of their parts are either too fast for the human eye (e.g., the trapping movements of carnivorous plants such as the Venus flytrap or bladderworts) or too slow (e.g., growth movements). Such movements are all the more fascinating because they can occur without joints and sliding parts by elastic deformation. This is also the case with the bird-of-paradise flower (*Strelitzia reginae*) (Figure 19). It is pollinated by birds that land on an upwardly open “perch” consisting of two fused purple petals. The weight of the bird pushes the perch downwards. This deformation causes the sides to fold outwards, exposing the stamens and pistil and allowing pollination [54].

#### 4.7.2. Biomimetic Product: Facade Shading System Flectofin^®^

**Figure 20:**
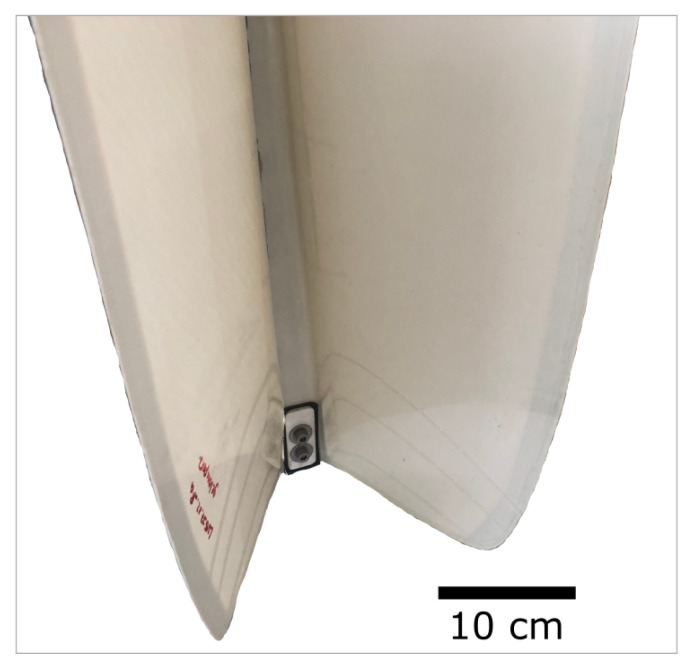
Detailed view of a demonstrator of the biomimetic Flectofin^®^ facade shading system. The more the backbone is bent, the more the two lamellae fold sideways.

The deformation mechanism of the bird-of-paradise flower was studied, abstracted, and technically implemented by biologists from the Botanic Garden Freiburg and architects and civil engineers from the University of Stuttgart. Like the biological model, the biomimetic product (Flectofin^®^) is a joint-free, infinitely adjustable, folding mechanism. It consists of a backbone and one or two lamellae, with the lamellae folding sideways, while the midrib bends (Figure 20). The Flectofin^®^ can be used wherever movable lamellae are required, such as in the shading of building facades [54,55].

#### 4.7.3. Frequently Asked Question: How to Recognize Bird-Pollinated Plants?

Many plant species have red or orange flowers that are particularly visible to birds. Since New World hummingbirds can hover freely in front of the flower, they do not need a perch to reach the nectar of the flowers, unlike Old World nectar and weaver birds. However, there are no flower-pollinating birds in Europe.

#### 4.7.4. Contributions to Sustainable Development

- Hinge-less compliant components contribute to the conservation of resources by reducing wear and tear and require little maintenance [56,57].
- Hinge-less components contribute to reduced waste generation as they are less susceptible to damage from deformation.
- The high aesthetics and functionality of the movement increase the appreciation of the biological models.

### 4.8. Flectofold—Curvilinear Folding

#### 4.8.1. Plant Model: The Waterwheel Plant

**Figure 21:**
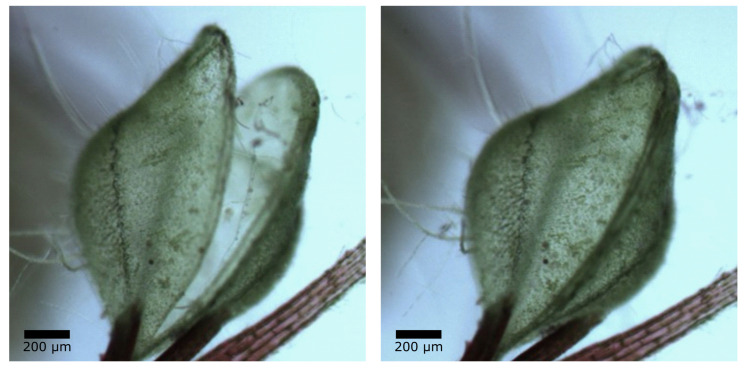
Traps of the carnivorous aquatic waterwheel plant *Aldrovanda vesiculosa* in an open state (**left**) and in a closed state (**right**). The two trap lobes are connected by a curved midrib (from [59]).

The hinge-less movement mechanism of the snap trap of the carnivorous waterwheel plant (*Aldrovanda vesiculosa*) has been studied by biologists from the Botanic Garden Freiburg. The underwater snap trap consists of two leaf halves with a curved midrib in-between (Figure 21). Morphological and anatomical studies, mechanical tests, and simulations have shown that the rapid movement of the traps is a combination of hydraulic turgor changes in the midrib zone, also known as the motor zone, and the release of pre-stresses in the tissues [58].

#### 4.8.2. Biomimetic Product: The Facade Shading System Flectofold

**Figure 22:**
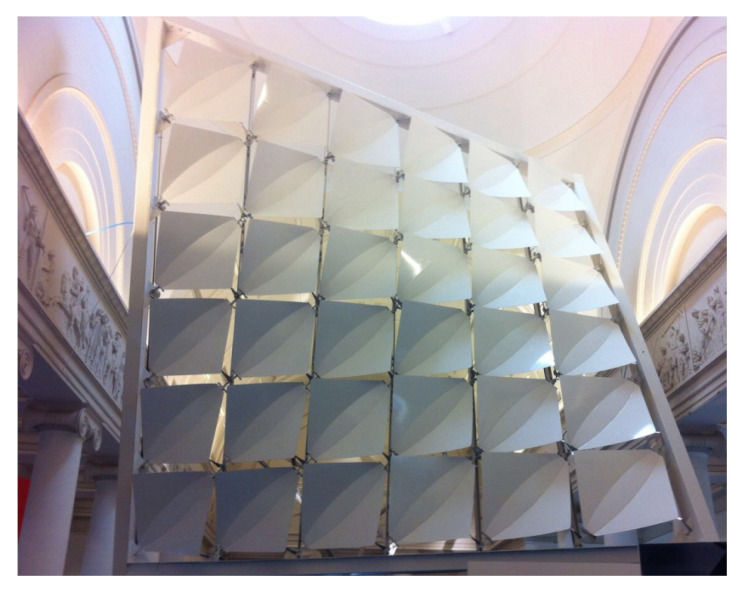
The demonstrator shows an application of Flectofold as a shading device. It consists of 36 Flectofold elements in an open state, each measuring 1 m × 1 m.

Inspired by the movement principles of the waterwheel snap trap, civil engineers and architects from the University of Stuttgart developed the Flectofold facade shading system. Figure 22 shows a demonstrator made up of 36 individual Flectofold elements. Each Flectofold consists of a midrib with adjacent curvilinear hinge zones and two wings, with the wings lifting as the midrib bends [60,61].

#### 4.8.3. Frequently Asked Question: What Is a Carnivorous Plant?

Although carnivorous plants derive their energy from photosynthesis, they also obtain additional essential nutrients from insects and other arthropods. Growing on nutrient-poor soils (e.g., low nitrogen), they have evolved traits to capture prey in traps and absorb nutrients from the killed and digested prey in order to grow and develop.

#### 4.8.4. Contributions to Sustainable Development

- Hinge-less compliant components contribute to resource conservation by reducing wear and tear [56,57].
- Hinge-less components contribute to reduced waste generation because they are less susceptible to damage from deformation.
- The high aesthetics and functionality of the movement increase the appreciation of the biological models.

### 4.9. Further Possible Stations

The biomimetic examples selected and presented here can be expanded to include a variety of additional stations for an outdoor educational trail about biomimetics in botanical gardens or parks. In the following list, we suggest possible additional stations, briefly describe them, and provide references:

- **Flying seeds and gliders—lightweight and auto-stable:**
  The self-stabilizing gliding flight of the Zanonia seed (*Alsomitra macrocarpa*) was the inspiration for the development of the manned flying wing glider by the aircraft designers I. Etrich and F. X. Wels [40]. Recent work on dandelion fruits has opened the way for novel plant-inspired micro-parachutes [62].
- **Shape-optimized components—growing like trees:**
  Physicist Claus Mattheck and his colleagues (Karlsruhe Institute of Technology, Germany) have developed a computer program called Computer Aided Optimization (CAO), which applies the principle of tree growth in order to optimize the shape of technical components [63].
- **Dragon tree and columnar cactus—branched lightweight constructions:**
  Inspired by the course of fibers in branched columnar cacti and dragon trees, modern braiding and knitting methods can be used to produce technical textiles with complex shapes [64,65,66].
- **Pomelo and coconut—damping and protecting:**
  Some fruits, such as pomelo and coconut, can withstand being dropped from over ten meters without damage because of their excellent shock-absorbing and puncture-resistant material structures. Inspired by the structure of their fruit walls, fiber-reinforced metal and polymer foams with puncture-resistant top layers have been developed for use in car shock absorbers and protective helmets [67,68,69].

## 5. Guided Tours about Biomimetics

Guided tours for interested people of all ages can be conducted along the educational trail about biomimetics. Depending on the length of the tour, certain stations can be selected, and the information on the panels (see Section 4) can be explained in more detail. On the one hand, visitors can take a closer look at the biological model. On the other hand, the biomimetic products can be shown to the visitors. Simple experiments can also be carried out to demonstrate the respective function of the biological model and the biomimetic product. In the following, we will present some ideas for simple experiments that have proven to be illustrative in recent years. Various construction instructions for simple models showing the deformation principle of the traps of carnivorous plants are presented in [70]. Self-actuated paper models showing the deformation principles of plant models applied to biomimetic products are described in [59,71].

### 5.1. Experiments Demonstrating the Self-Cleaning Function

**Figure 23:**
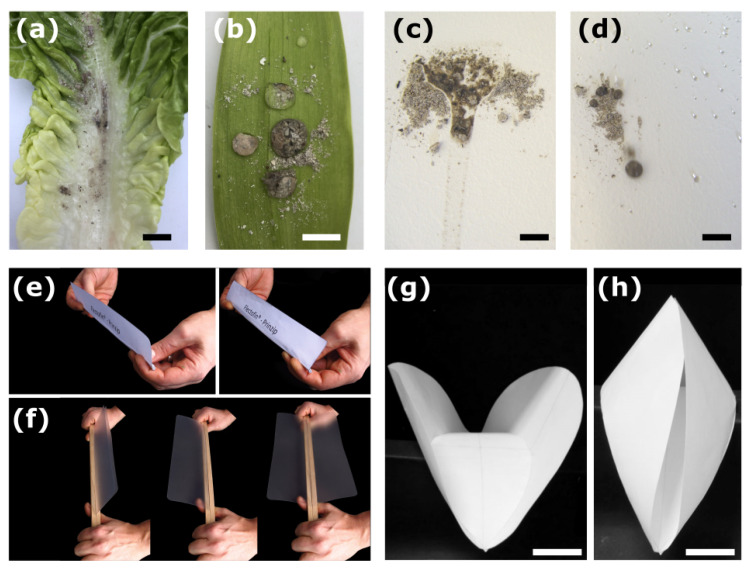
Simple experiments using biological models and biomimetic products. (**a**) Lettuce leaf without self-cleaning function. (**b**) Tulip leaf with self-cleaning function. Sample plates coated with paint (**c**) without and (**d**) with the Lotus-Effect^®^. (**a**,**c**) On the surfaces without the self-cleaning function, the water droplets spread and scatter the ash on the surface. (**b**,**d**) In contrast, the ash from the self-cleaning surfaces can be easily washed off with a few drops of water. Scale bars in (**a**–**d**) equal 1 cm. Hands-on demonstrator of the Flectofin^®^ composed of (**e**) paper or (**f**) a wooden backbone and transparent plastic foils. Self-actuated paper-based models of the snap trap of the waterwheel plant in (**g**) an open state and (**h**) a closed state (from [59]). Scale bars in (**g**,**h**) equal 4 cm.

To demonstrate the self-cleaning effect, plant leaves, samples coated with facade paint, chimney ash, and a spray bottle filled with water are all that are needed. Plant leaves with the self-cleaning effect (e.g., lotus, tulip, charcoal, or marigold) and without it (e.g., water lily, lettuce) can be collected in the botanical garden. Test kits with sample panels coated with the self-cleaning facade paint Lotusan^®^ and with a non-self-cleaning facade paint can be ordered free of charge from the Internet (available at www.sto.de). The plant leaves and the sample plates are sprinkled with ash and then rinsed with the water from the spray bottle (Figure 23a–d). The spherical water droplets easily wash off the ash from the self-cleaning biological and artificial surfaces. On the non-self-cleaning surfaces, however, the water spreads and the ash can only be washed off with much water under pressure.

### 5.2. Experiments Demonstrating the Deformation Principle of the Flectofin^®^

Simple physical demonstrators show the underlying deformation principle of both the biological model and the facade shading system Flectofin^®^, namely so-called lateral–torsional buckling (Figure 23e,f). The demonstrators consist of a backbone and one lamella or two lamellae (double-Flectofin^®^). Bending the backbone causes the attached lamella(e) to deflect sideways up to 90°, which is equivalent to shading the facade. Detailed construction instructions for single- and double-Flectofin^®^ demonstrators either made of paper or composed of a plastic or wooden rod as the backbone and polymer foils as the lamellae can be found in [72].

### 5.3. Experiments Demonstrating the Deformation Principle of the Flectofold

Because the snap traps of the waterwheel plant are microscopically small, they cannot be directly observed by the garden visitors. Nevertheless, the functional principle can be explained using paper models (Figure 23g,h). Detailed construction instructions for both the snap traps of the biological model and the biomimetic facade shading system Flectofold are published in [71]. The snap trap model consists of a midrib connected to the two lateral flaps by curved lines. The paper-based models are self-actuating by the drying of the wet actuating layer glued to the surface of the resistance layer, whose fibers are aligned perpendicular to each other. The Flectofold demonstrator, however, consists of a squared polymer sheet divided into a lenticular midrib with an actuating multi-layer veneer composite and two triangular wings. The construction instructions for the Flectofold demonstrator are given in [71]. Bending of the midrib results in a hoisting of the plant flaps or artificial wings. A straight unbent midrib, therefore, corresponds to the shading of the facade.

## 6. Discussion and Future Prospects

Botanical gardens, zoological gardens, and natural history museums are scientific collections, in addition to being places of education, teaching, and research [73]. Moreover, the collections in natural history museums contain millions of specimens representing 12% of total global biodiversity [74] and making species from remote areas or fossil records accessible to the general public [75,76]. Given the increasing loss of global biodiversity, collections will become even more important in the future. Estimates of the number of living species vary widely, with numbers ranging between 3 to over 110 million species having been extrapolated from the 1.75 million species described in the mid-1990s [75]. According to Mora et al. [77], approximately 11 million species live on Earth today, of which about 2.0 million species have been scientifically described [78]. This includes about 10 million species of animals, about 320,000 species of plants, and about 620,000 species of fungi and lichens. Microorganisms probably account for most of the world’s biodiversity with an estimated number between 1.5 and 2.0 billion. However, their numbers are difficult to estimate and are, therefore, not properly represented in the estimate [77].

In recent years, botanical gardens, zoos, and museums have often proven to be places of innovative research, including in modern fields such as biomimetics. The biodiversity found in these locations provides scientists with a wealth of inspiration for successful innovative developments. More than 90 botanical gardens and about 850 zoos and animal parks are open to the public in Germany, with more than 1800 botanical gardens being accessible worldwide. Botanical gardens cultivate about 90,000 to 100,000 plant species, representing about 20% of known plants. The proportion of animal species kept in zoos is much smaller, but almost all species are present in natural science museums, although not all are always accessible to the public. Unfortunately, in times of economic constraints, botanical gardens, museums, and zoos themselves are increasingly threatened, with incalculable consequences for research, teaching, and public education [75].

Natural scientific collections have a long tradition. Humans originally collected and cultivated plants for food and medicine and formed them into utensils. Over time, this led, for example, to the creation of monastic herb gardens and, later, to gardens enriched with exotic plants that increasingly served the representative purposes of the nobility and the bourgeoisie and, finally, to the multitude of botanical gardens of today. The diversity of objects collected in natural history museums, encompassing most of nature, both animate and inanimate, and especially the living collections in botanical and zoological gardens offer tremendous opportunities for biomimetic research and hark back to the original function of collections: the use of natural resources. The wide range of adaptations to various environmental conditions, of design principles, and of material combinations from living nature represents an immense potential for biomimetic innovation in technology today and in the future [73,74,79].

## 7. Conclusions

Over the centuries, botanical gardens have changed their focus from the representation of a paradise on Earth, to gardens specializing in medicinal, poisonous, and herbal plants studied by students of medicine or pharmacy at universities, to places of lifelong learning, research, and relaxation for students, and other interested parties of all ages. In recent years, learning from living nature with the aim of developing technical applications has played a special role in botanical gardens, as reflected in educational trails and guided tours about biomimetics. In this publication, we provided text and images for eight panels of a biomimetics trail, plus information on simple experiments as part of such a guided tour. Each botanical garden can add further stations to the trail. Both the educational trail and the guided tour concerning biomimetics will lead to an understanding of the functions and underlying functional principles transferred from the biological model to the technical application and will demonstrate the contribution of the biomimetic product to sustainable development. In this context, the view of sustainable development can be brought into focus, as a biological model does not necessarily guarantee a contribution to sustainability [26] (see the example of barbed wire with its negative social impacts). Instead, a sustainable product only emerges when a particular ethos and respectful treatment of nature complement the technological ambitions of the practice [30] (see self-cleaning and self-repairing functions in artificial products).

## Figures and Tables

**Table 1:** List of botanical gardens with an educational trail or other garden-related educational offers with a focus on biomimetics or biomimicry (in alphabetical order of city).

City, Country	Botanical Garden and Other Places	Portfolio	Reference
Bocholt, Germany	Campus Bocholt of the Westfälische Hochschule	Educational trail, guided tours	[16]
Cambridge, United Kingdom	Cambridge University Botanic Garden	Educational trail, publications	[17]
Darmstadt, Germany	Botanical Garden of the Technical University of Darmstadt	Educational modules	[18]
Dresden, Germany	Botanical Garden of the Technical University Dresden	Educational trail, guided tours, lectures, publications, teaching modules	[19]
Freiburg, Germany	Botanic Garden of the University of Freiburg	Educational trail, guided tours, lectures, publications, teaching modules	[20]
Konstanz, Germany	Botanical Garden of the University of Konstanz	Teaching material	[21]
Melbourne, Australia	Royal Botanic Gardens Victoria	Education and training program	[22]
Tivon, Israel	Botanical Garden at Oranim College	Biomimetic path	[23]

## Data Availability

Not applicable.
